# A Complex Presentation of Acute Postoperative Negative-Pressure Pulmonary Edema: A Case Report and Review of Literature

**DOI:** 10.7759/cureus.42152

**Published:** 2023-07-19

**Authors:** Larri Rudman, Javier B Chambi-Torres, Farah Chohan, Mohammad Aftab, Xinyu Cao, George Michel

**Affiliations:** 1 Internal Medicine, Larkin Community Hospital, South Miami, USA; 2 Research and Academic Affairs, Larkin Community Hospital, South Miami, USA; 3 Pulmonary and Critical Care, Larkin Community Hospital, South Miami, USA; 4 Pulmonary and Critical Care Medicine, Larkin Community Hospital Palm Springs Campus, Hialeah, USA

**Keywords:** fluid rescusitation, upper airway obstruction, negative inspiratory force, laryngospasm, postoperative pulmonary edema, negative-pressure pulmonary edema

## Abstract

Negative-pressure pulmonary edema (NPPE) is an uncommon diagnosis that requires a high clinical suspicion to recognize and manage and has high morbidity and mortality. It usually results secondary to markedly negative intrapleural pressure due to the forceful inspiration against the obstructed airway from upper airway infection, tumor, or laryngospasm. We present a case of a 27-year-old female with morbid obesity who underwent sleeve gastrectomy and developed NPPE upon emergence from anesthesia. The focus of supportive care should be on addressing the obstruction in the upper airway through either endotracheal intubation or cricothyroidotomy. Additionally, it is important to initiate lung-protective positive-pressure ventilation and promote diuresis, unless the patient is in a state of shock. The resolution of pulmonary edema is typically swift, partially due to the preservation of alveolar fluid clearance mechanisms. In the literature review, we delve into the clinical presentation, pathophysiology, and management of NPPE or post-obstructive pulmonary edema.

## Introduction

Pulmonary edema, which occurs due to sudden airway obstruction, poses a significant risk to life. It manifests swiftly and unexpectedly in individuals who are otherwise in good health. Pulmonary edema can be broadly classified as cardiogenic and non-cardiogenic (including acute respiratory distress syndrome (ARDS), high-altitude pulmonary edema (HAPE), neurogenic pulmonary edema, toxic inhalation pulmonary edema, and post-obstructive pulmonary edema (POPE) [1]. Additional less common causes of non-cardiogenic pulmonary edema include allergic reactions (e.g., anaphylaxis), transfusion-related acute lung injury (TRALI), and certain medications or drug overdoses. It is worth noting that these classifications are not mutually exclusive, and some cases of pulmonary edema may have mixed causes or overlap between the different types. A thorough medical evaluation is necessary to determine the underlying cause and guide appropriate treatment. There are two types of POPE. POPE I is the result of a sudden, acute upper airway blockage. POPE II develops after surgical treatment of chronic upper airway blockage [2]. Treatment for both is usually supportive [3]. With proper management, complete and rapid recovery can be anticipated. 

## Case presentation

**Figure 1:**
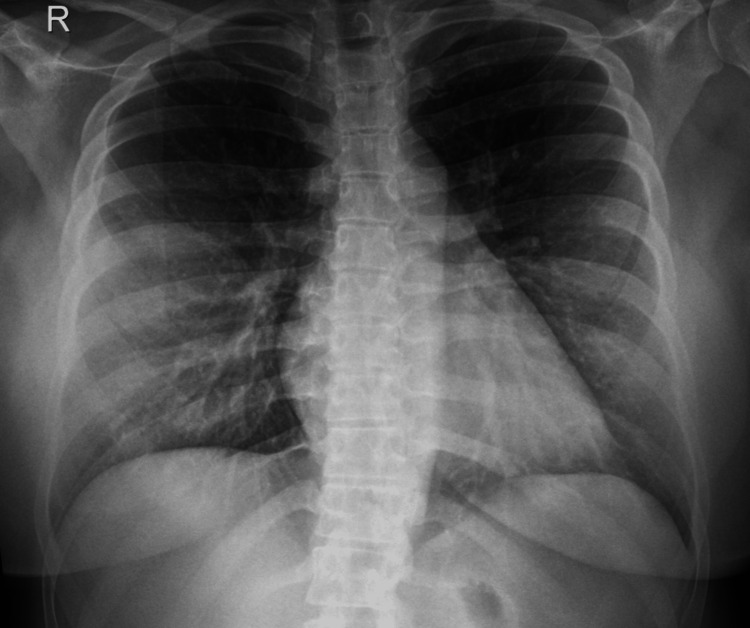
Pre-operative chest X-ray with unremarkable findings

 A 27-year-old female with a medical history that is significant only for obesity (Class II) and past surgical history important for liposuction and breast augmentation was admitted for elective laparoscopic sleeve gastrectomy. Preoperative clinical examination revealed an American Society of Anesthesiologists (ASA) II based on the patient's BMI of 35.6 kg/m^2^ without other comorbidities and an unremarkable preoperative chest X-ray (Figure 1). The patient has no documented history of pulmonary disease or postoperative complications.

Induction premedication was achieved with IV midazolam 2 mg and succinylcholine 70 mg with a successful intubation with a cuffed 7.5 cm endotracheal tube. Throughout the procedure, maintenance of anesthesia was achieved with continuous propofol (200 mg) and fentanyl (100 mcg initially followed by two subsequent doses of 50 mcg), and neuromuscular blockade was achieved with rocuronium 50 mg. Intraoperative monitoring with continuous ECG, pulse oximetry, and blood pressure measurement was within normal limits during the two-hour and 15-minute long surgery, throughout which the patient was administered 1300 ml balanced crystalloids with 300 ml of urine output and 50 ml of estimated blood loss. No intraoperative complications were reported. Following the completion of the laparoscopic sleeve gastrectomy, the patient underwent a reversal of neuromuscular blockade with sugammadex. The patient demonstrated head lift, spontaneous ventilation, and protective reflexes. An attempt to extubate the patient was made, with the cuff deflated, there was no evidence of strider, other signs airway obstruction. However, soon after reversal of neuromuscular blockade and immediately prior to extubation, the patient desaturated into the high 70s-low 80s with assoicated tachycardia in the 130s, tachynea with respiratory rate (RR) in the high 30s-40s.

**Figure 2:**
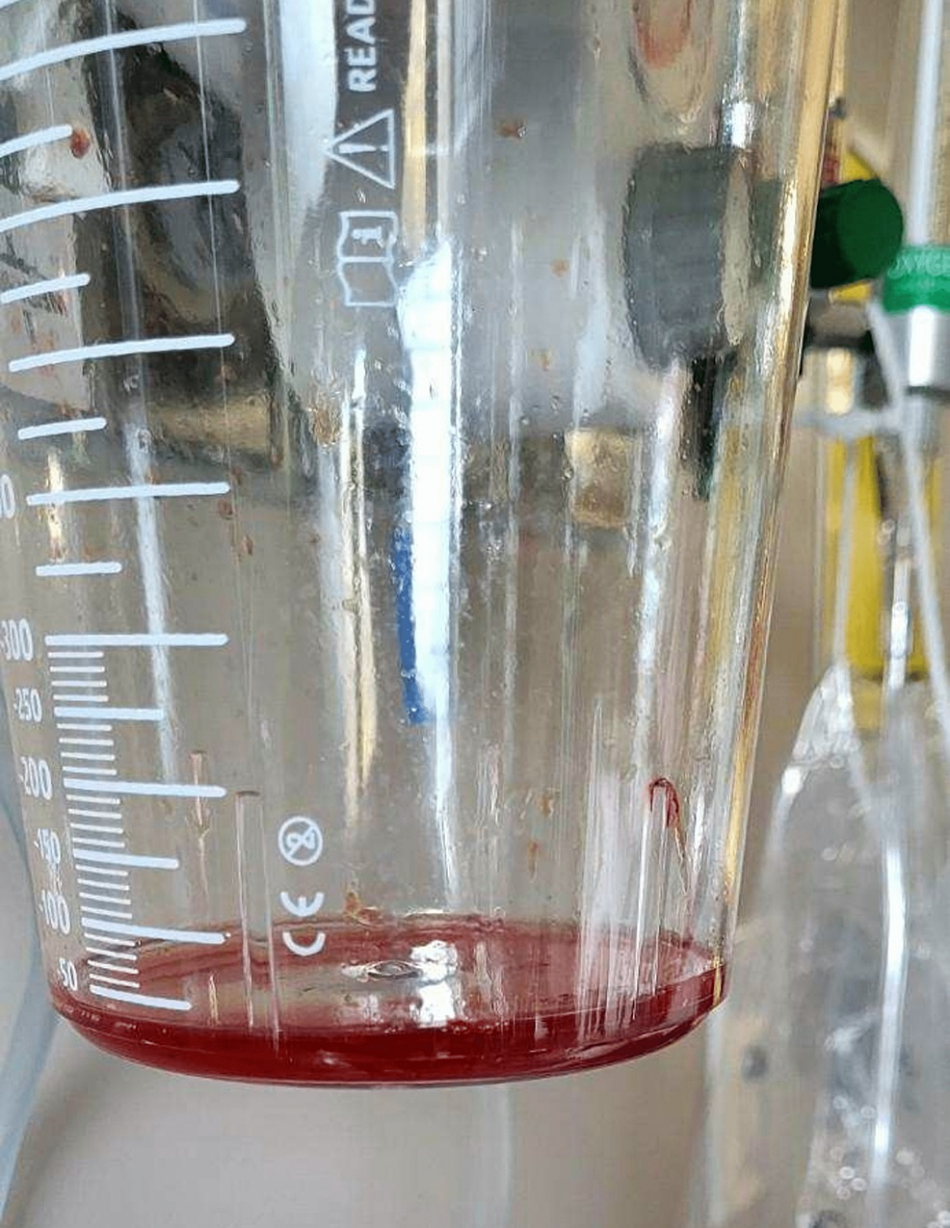
Post-suction pink frothy sputum suggestive of pulmonary edema

Upon desaturation, positive pressure ventilation was resumed, and frothy pink sputum was noted coming out of the endotracheal tube (ETT). On further examination, the jugular veins were found to be flat. Cardiac auscultation was normal without any added sounds. Bilateral end-inspiratory crepitations were heard bilaterally. Since the patient had no prior history of hypertension and congestive heart failure (CHF), based on the presentation (rapid onset, acute hypoxemia, frothy pink sputum), negative-pressure pulmonary edema (NPPE) was suspected, and furosemide 30 mg intravenous pyelogram (IVP) was administered with continuous suctioning of the airway. The patient had immediate diuresis of 300 ml. Still, with ongoing suctioning of frothy pink sputum (Figure 2), 10 mg of furosemide IV was administered in addition to 50 mcg of epinephrine. The patient's vital signs stabilized; afebrile, pulse rate 80, RR 15, blood pressure 107/77, and pulse oximetry returned 100%.

**Figure 3:**
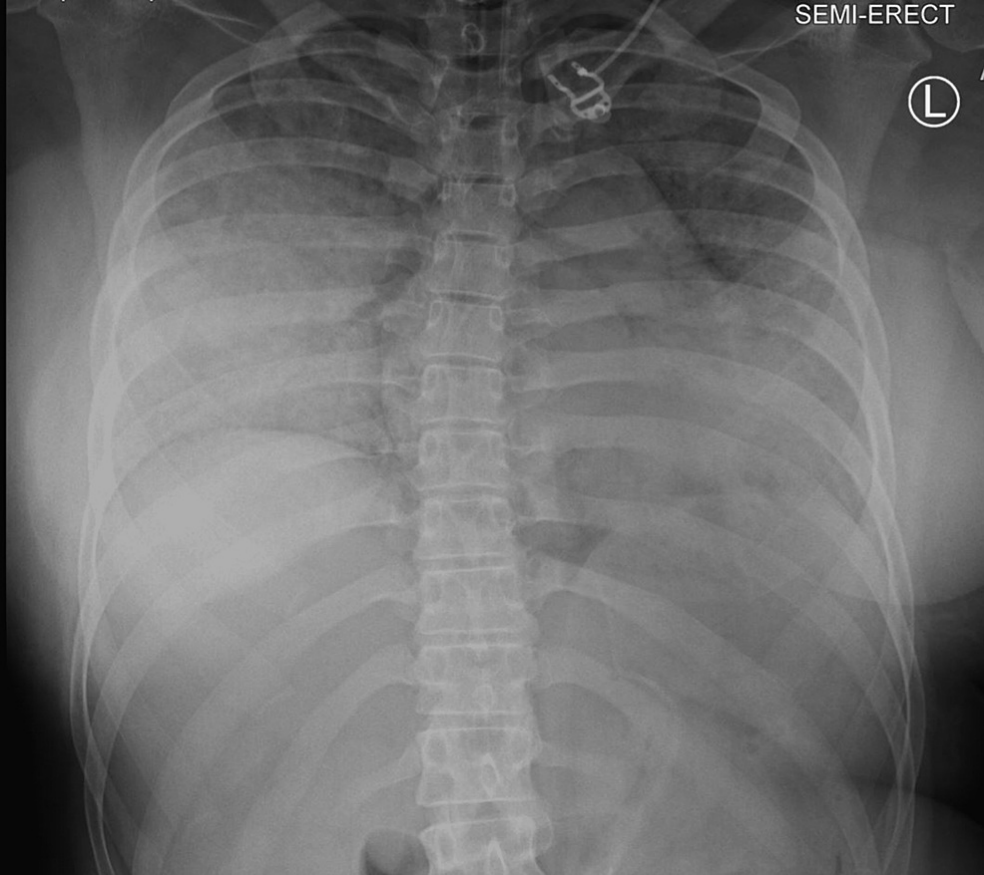
Diffuse bilateral confluent ground-glass attenuation throughout the lungs. Findings compatible with diffuse alveolar edema/ARDS. ARDS: acute respiratory distress syndrome

**Figure 4:**
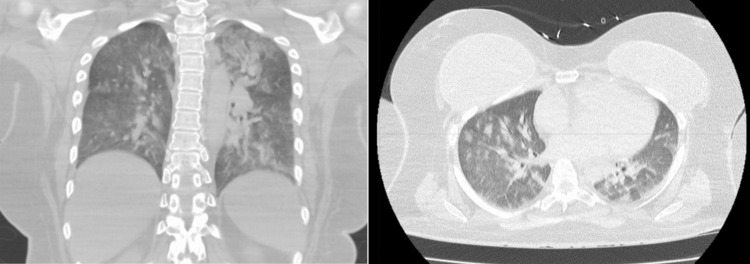
CT chest showing diffuse, bilateral ground-glass and consolidative opacities, peri broncho vascular wall thickening, and tree-in-bud opacities.

A decision was made to keep the patient intubated. IV midazolam 2 mg and rocuronium 50 mg were administered, and the patient was subsequently transferred to the ICU for further management. The ventilatory settings at that time were: Pressure controlled with positive end-expiratory pressure (PEEP) 10, peak inspiratory pressure (PIP) 30, RR 15, fraction of inspired oxygen (Fi02) 100%, tidal volume (TV) 500 ml. Upon admission to the ICU, the patient's ventilator settings were adjusted to assist control/volume control (AC/VC)+ RR 20 bpm, TV 420 ml, FiO2 40%, PEEP 10. An arterial blood gas (ABG) test was obtained and it showed pH 7.33, partial pressure of carbon dioxide (PCO2) 43 mmHg, and partial pressure of oxygen (PO2) 160 mmHg. Chest x-ray performed following desaturation revealed diffuse infiltrates consistent with pulmonary edema (Figure 3). Cross-sectional thoracic imaging showed diffuse, bilateral ground-glass and consolidative opacities, peri broncho vascular wall thickening, and tree-in-bud opacities (Figure 4).

**Figure 5:**
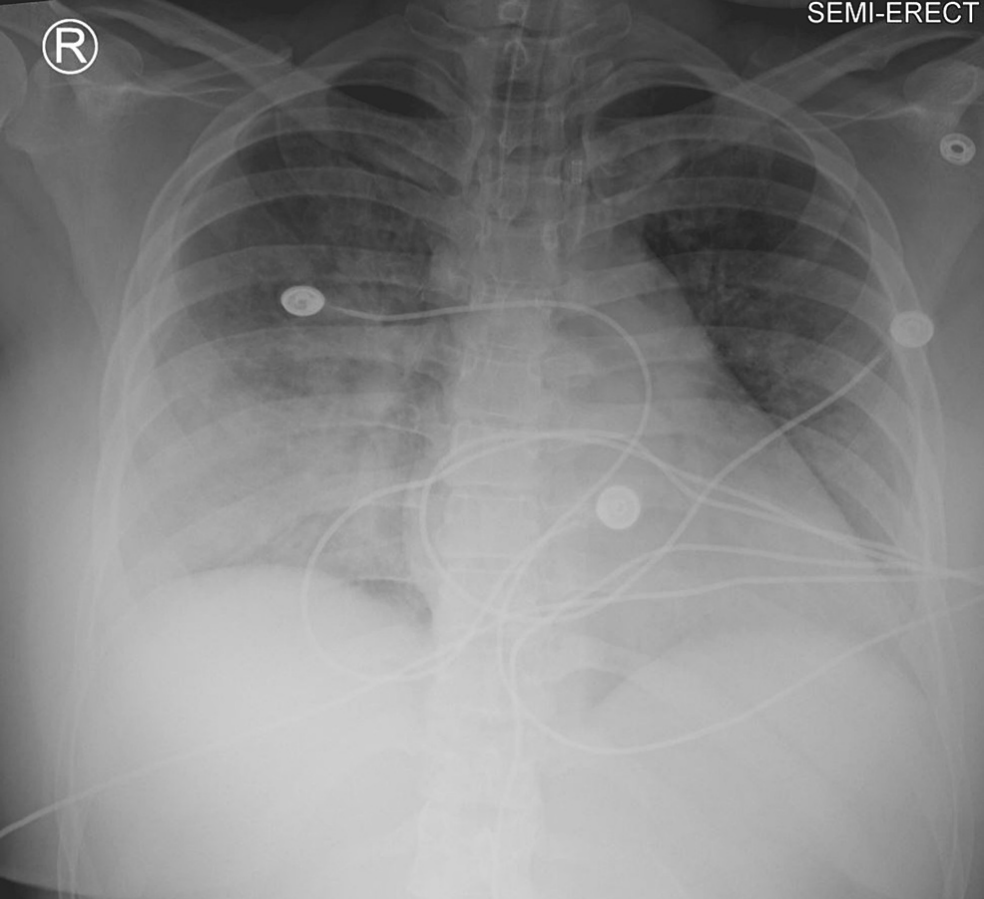
Chest X-ray obtained following diuresis. Redemonstration of diffuse pulmonary edema pattern with mild interval improvement compared to prior radiograph.

The patient's brain natriuretic peptide (BNP) markedly increased from 92 pg/mL to 1220 pg/mL. Based on radiographic and lab findings, the patient was started on Lasix IV 40 mg with a urine output of 1.8 L. The patient was maintained on the ventilator for approximately 24 hours. A follow-up chest X-ray demonstrated mild interval improvement in the diffuse bilateral confluent opacities (Figure 5).

Before the spontaneous breathing trial, the ventilator settings were: AC/VC+, RR 20 bpm, TV 420 ml, FiO2 40%, PEEP 10 with ABG showing a pH of 7.35, pCO2 46 mmHg, pO2 96 mmHg. While on spontaneous breathing trial with pressure support 10 cmH2O, FiO2 40%, PEEP 5 cmH2O, the patient's ABG demonstrated pH 7.43, pCO2 36 mmHg, pO2 139 mmHg, and she was successfully extubated to heated high flow nasal cannula, which was well tolerated. Supplemental oxygen was titrated overnight and the patient was liberated from all modes of supplemental oxygenation. In light of the patient meeting discharge criteria from a surgical standpoint and no further cardiopulmonary complications, the patient demonstrated convalescence and was thus discharged. 

## Discussion

NPPE occurs when acute pulmonary edema and respiratory distress develop after attempting to breathe against an obstructed upper airway. The most common cause in adults is laryngospasm, representing 50% of NPPE cases [4], while upper respiratory infections are the most common cause in children [3]. According to the literature, NPPE occurs in the surgical setting of general anesthesia; the pulmonary edema develops within a few minutes after the upper airway obstruction, and its incidence is about 0.05-0.1% in the anesthesia setting [5-6]. In addition, the frothy pink fluid observed in the endotracheal tube, like in our patient, has also been described [7].

POPE requires rapid intervention and may be confused with other causes of postoperative respiratory distress. Although symptoms typically appear one hour after the inciting incident, delayed onsets have been documented [8-11]. Signs and symptoms, including agitation, tachypnea, tachycardia, frothy pink pulmonary secretions, rales, and progressive oxygen desaturation, point towards diagnosing POPE in the appropriate clinical setting. Chest radiograph findings of pulmonary edema support the diagnosis. However, other causes of pulmonary edema should also be considered [12]. The likelihood of a POPE diagnosis is increased by a history of normal cardiac function and, in particular, the symptoms mentioned above in a relatively young person.

There are two proposed pathophysiological mechanisms of NPPE [4]. The first occurs following significant diaphragmatic effort, which is generated to overcome the obstructed upper airway during inspiration. Following the physiology of respiration, inspiration generates an increased venous return, which is exaggerated by the notably increased negative pleural pressure, resulting in higher right-sided cardiac volumes and subsequent increased hydrostatic pressure in the pulmonary vessels [3]. This increases the alveolar-capillary gradient, disrupts the alveolar-capillary membrane, and extravasates fluid into the alveoli. The second mechanism suggests an increased respiratory effort could prompt the damage of the alveolar epithelium and pulmonary capillary membranes, facilitating fluid passage into the alveoli [3]. The intra-alveolar fluid is most likely transudate produced by a hydrostatic mechanism, according to a fluid analysis of 10 patients [13].

We hypothesize that two possible causes or triggers of NPPE in our patient were laryngospasm and her morbid obesity history. Laryngospasm is the most common cause of NPPE in adults, and it could be triggered by contact between the tracheal tube and the throat. In addition, obese patients have smaller laryngeal areas, which produces more negative pressure during inspiration [14]. The smaller laryngeal area of our patient could trigger the intensity of friction of the tracheal tube with the tissue with the subsequent muscle spasm.

Our patient received general anesthesia, rocuronium for the neuromuscular blockade, and sugammadex for rocuronium-induced neuromuscular blockade reversal postoperatively. Sugammadex is a modified gamma-cyclodextrin approved by the FDA in adults for antagonism of non-depolarizing neuromuscular blockade agents (NMBAs) like rocuronium and vecuronium [15]. It encapsulates the free NMBA in plasma, thus decreasing the plasma concentration, and inducing the passage of free NMBA from the muscle to the plasma, leaving free nicotinic receptors at the neuromuscular junction [16]. The reported side effects of sugammadex are nausea, vomiting, headache, itching, procedural pain, dysgeusia [16], and bradycardia [10]. We found case reports that described NPPE development after sugammadex administration in adults (Table 1) [17-21], like in our case. Most of these cases, similar to ours, had tracheal intubation as the airway technique, rocuronium as NMBA during surgery, and laryngospasm as the possible cause of NPPE. However, ages were diverse, ranging from 17-76 years. 

**Table 1:** Case reports on development of NPPE (rocuronium used as NMBA in all of these cases) M: male; F: female; NMBA: non-depolarizing neuromuscular blockade agent; TI: tracheal tube; ETT: endotracheal tube; LMA: laryngeal mask airway; NPPE: negative pressure pulmonary edema

Author	Gender	Age (years)	BMI (kg/m2)	Medical history	Airway technique	Type of surgery	Possible cause of NPPE	NMBA used during surgery
Suzuki et al [17]	M	41	26.3	Asthma	TI	Laparoscopic appendectomy	Laryngospasm	Rocuronium
Lee et al [18]	F	17	22	Papillary thyroid cancer removal	TI	Lateral neck node dissection	Laryngospasm	Rocuronium
Han et al [19]	M	25	22.1	Allergic rhinitis	TI	Testis repair	Laryngospasm	Rocuronium
Choi et al [20]	M	25	27	Unremarkable	TI	Bilateral orchiopexy	Remifentanil-induced muscle rigidity	Rocuronium
Kao et al [21]	M	46	21.7	Asthma	TI	Functional endoscopic sinus surgery	Laryngospasm	Rocuronium
	F	29	23.1	Unremarkable	TI	Nasal surgery	ETT obstruction by biting	Rocuronium
Current case	F	27	35	Morbid obesity	TI	Laparoscopic sleeve gastrectomy	Possible laryngospasm, and increased neck circumference.	Rocuronium

Reversing the airway obstruction, increasing the oxygen saturation, and diuretic therapy compose the primary treatment of NPPE [22]. Additionally, due to its advantageous effects on gas exchange, alveolar unit recruitment, prevention of pulmonary edema caused by hydrostatic forces, and preservation of airway patency, PEEP is widely utilized in patients receiving mechanical ventilation. This coincides with the management that our patient received through airway suction and oxygen therapy, which produced immediate correction of oxygen saturation. Still, the diuretic failed to resolve the pulmonary edema according to the initial post-treatment images.

## Conclusions

Acute postoperative NPPE is a rare cause of non-cardiogenic pulmonary edema, with laryngospasm as the most common cause in adults. Obesity could be a factor that triggers laryngospasm because of the smaller pharyngeal area and the increased contact between the tracheal tube and the tissue. Our patient was managed appropriately and without subsequent sequelae. Treatment is usually supportive. With proper management, complete and rapid recovery can be anticipated.
